# Clinical Factors Predictive for Intracranial Hemorrhage in Mild Head Injury

**DOI:** 10.1155/2017/5385613

**Published:** 2017-11-20

**Authors:** Chaiyaporn Yuksen, Yuwares Sittichanbuncha, Jayanton Patumanond, Sombat Muengtaweepongsa, Kasamon Aramvanitch, Amornrat Supamas, Kittisak Sawanyawisuth

**Affiliations:** ^1^Department of Emergency Medicine, Faculty of Medicine Ramathibodi Hospital, Mahidol University, Bangkok 10400, Thailand; ^2^Clinical Epidemiology Unit and Clinical Research Center, Faculty of Medicine, Thammasat University, Pathum Thani 12120, Thailand; ^3^Department of Medicine, Faculty of Medicine, Thammasat University, Pathum Thani 12120, Thailand; ^4^Department of Medicine, Faculty of Medicine, Khon Kaen University, Khon Kaen 40002, Thailand; ^5^Sleep Apnea Research Group, Research and Training Center for Enhancing Quality of Life of Working-Age People, Research Center in Back, Neck, Other Joint Pain and Human Performance (BNOJPH), Khon Kaen University, Khon Kaen 40002, Thailand

## Abstract

Patients with mild head injuries, a GCS of 13–15, are at risk for intracranial hemorrhage. Clinical decision is needed to weigh between risks of intracranial hemorrhage and costs of the CT scan of the brain particularly those who are equivocal. This study aimed to find predictors for intracranial hemorrhage in patients with mild head injuries with a moderate risk of intracranial hemorrhage. We defined moderate risk of mild head injury as a GCS score of 13–15 accompanied by at least one symptom such as headache, vomiting, or amnesia or with alcohol intoxication. There were 153 patients who met the study criteria. Eighteen of the patients (11.76%) had intracranial hemorrhage. There were four independent factors associated with intracranial hemorrhage: history of hypertension, headache, loss of consciousness, and baseline GCS. The sensitivity for the presence of intracranial hemorrhage was 100% with the cutoff point for the GCS of 13. In conclusion, the independent factors associated with intracranial hemorrhage in patients with mild head injury who were determined to be at moderate risk for the condition included history of hypertension, headache, loss of consciousness, and baseline GCS score.

## 1. Introduction

Head injury is a common presentation at the Emergency Room (ER). In the USA, approximately 1.4 million patients had head injuries in 2010. Of those, 275,000 patients required hospital admission and 52,000 patients suffered mortality, which came at a total cost of more than 60 million USD [1]. Head injury can be classified into three categories according to the Glasgow Coma Scale (GCS): mild (GCS 13–15), moderate (GCS 9–12), and severe (GCS < 8). Mild head injury is the most common and accounts for 80% of all head injury cases at the ER [2]. The main issue in mild head injury cases is early detection of intracranial hemorrhage. In recent studies, only 15% of patients with mild head injuries patients had abnormal brain imaging and only 1% of patients required brain surgery [3, 4].

Several reports have shown that patients with mild head injuries can be categorized as being at high, moderate, or low risk for intracranial hemorrhage [3, 5–7]. Those who are at high risk require computed tomography (CT) of the brain. The characteristics of these patients include presence of seizure or neurological deficits. For those with low risk or who were asymptomatic with a GCS of 15, no further investigation is required. The rest of the patients are considered to be at moderate risk for intracranial hemorrhage; a CT scan of the brain is warranted in selected cases. Clinical decision is needed to weigh between risk of intracranial hemorrhage and cost/risk of the CT scan of the brain. This study aimed to find predictors for intracranial hemorrhage in patients with mild head injuries with a moderate risk of intracranial hemorrhage.

## 2. Methods

This was a retrospective analytical study conducted at the ER in Ramathibodi Hospital in Bangkok, Thailand. The study period was between September 1, 2013, and August 30, 2016. The inclusion criteria were mild head injury, moderate risk for intracranial hemorrhage, age between 15 and 59 years, and having had a CT scan of the brain. We defined moderate risk of mild head injury as a GCS score of 13–15 accompanied by at least one symptom of the following: headache, vomiting, or amnesia or with evidence of alcohol intoxication. Patients with mild head injuries without any symptom or accompanied by seizures, neurological deficit, or evidence of skull fracture were excluded.

Operational definitions of studied symptoms were as follows: syncope was defined as sudden loss of consciousness less than 15 minutes (usually less than five minutes); the transient loss of consciousness was defined by loss of consciousness over 15 minutes or observed/witnessed loss of consciousness; headache was any degree of headache after head injuries; vomiting was after head injuries less than two times; amnesia was a deficit in short term memory [4].

Clinical data of all eligible patients were recorded including baseline characteristics, triage level at presentation, comorbidities, and medications, as well as causes, symptoms, and characteristics of the head injury. The triage level was assessed by staff at the ER and classified according to five levels (0–5) [8]. The outcome of the study was any intracranial hemorrhage detected by CT scan of the brain including epidural hematoma, subdural hematoma, subarachnoid hemorrhage, intracerebral hemorrhage, and cerebral contusion. The CT scan of the brain was performed within two hours after an ER visit following the standard procedure of the hospital. These CT findings were officially reported by an attending radiologist.

*Statistical Analysis*. Eligible patients were categorized as being either with or without intracranial hemorrhage. Clinical comparisons between the two groups were compared using descriptive statistics. Risk factors for intracranial hemorrhage were analyzed using univariate and multivariate logistic regression analyses. Those factors with a *p* value of less than 0.20 according to univariate logistic analysis were included in the multivariate logistic analysis. The goodness of fit of the multivariate logistic analysis model was evaluated using the Hosmer-Lemeshow test. Results of the model were reported as adjusted odds ratio and 95% confidence interval (CI). The cutoff points of independent predictors for intracranial hemorrhage that were numerical factors were evaluated using the receiver operating characteristic (ROC) curve. The entire analysis was performed using STATA software, version 10.1 (College Station, Texas, USA).

## 3. Results

During the study period, there were 153 patients who met the study criteria. Eighteen of the patients (11.76%) were found to have intracranial hemorrhage. There were two significant factors found to be associated with intracranial hemorrhage: the GCS and proportion of hypertension (Table 1). The intracranial hemorrhage group had a lower mean GCS (14.39 versus 14.85; *p* value < 0.001) and higher proportion of patients with hypertension (22.22% versus 6.67%; *p* value 0.049) than those without intracranial hemorrhage.

Regarding symptoms of head injury, a significantly higher proportion of patients with intracranial hemorrhage experienced syncope (27.78% versus 8.89%), headache (50.00% versus 24.63%), and loss of consciousness (77.78% versus 26.67%) than those without intracranial hemorrhage (Table 2). There were no statistical differences in terms of causes or characteristics of the head injuries (Table 2).

**Figure 1:**
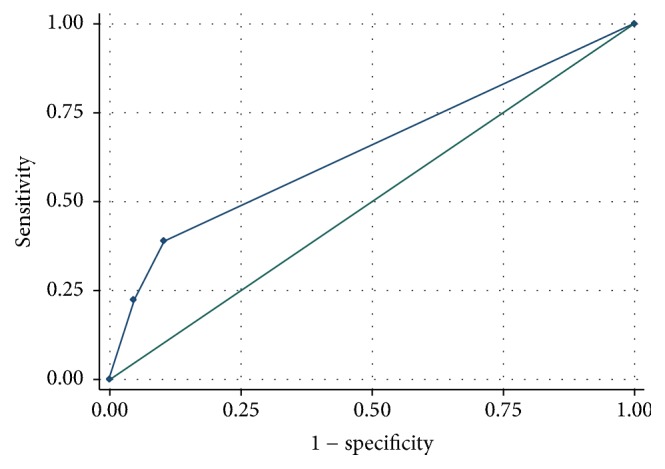
A receiver operating characteristic (ROC) curve of Glasgow Coma Scale and intracranial hemorrhage in patients with moderate risk of mild head injuries.

There were four independent factors associated with intracranial hemorrhage including history of hypertension, headache, loss of consciousness, and baseline GCS (Table 3), the adjusted odds ratios (95% CI) of which were 11.376 (1.317, 98.262), 4.011 (1.097, 14.661), 10.282 (2.436, 43.395), and 0.164 (0.057, 0.474), respectively. The Hosmer-Lemeshow chi square statistic was 2.93 with a *p* value of 0.891. The cutoff point for a GCS score of 14 had sensitivity of 77.78% and specificity of 4.44% for intracranial hemorrhage with an area under the ROC curve of 64.55% (Figure 1). The sensitivity increased to 100% with the cutoff point for the GCS of 13.

## 4. Discussion

After adjustment for comorbidities, cause of head injury, GCS, symptoms of head injury, four independent factors associated with presence of intracranial hemorrhage in patients with mild head injuries who were at moderate risk for the condition, namely, history of hypertension, headache, loss of consciousness, and baseline GCS score.

Headache and loss of consciousness were the symptoms/signs of head injury that were suggestive of intracranial hemorrhage, but syncope and amnesia were not (Table 3). Although headaches in mild head injury patients may stem from a number of different causes (such as head contusion), they may indicate a fourfold greater risk for intracranial hemorrhage. Similarly, according to a study out of Spain, even mild headaches can double the risk of intracranial hemorrhage (adjusted odds ratio of 2.19 with 95% CI of 1.19–4.03) [9]. Loss of consciousness has also been reported as a significant risk factor for intracranial hemorrhage in cases of mild head injury [10, 11]. As previously reported, the dangerous mechanism indicating intracranial hemorrhage are pedestrian struck by a motor vehicle, an occupant ejected from a motor vehicle, or a fall from an elevation of three or more feet or five stairs [4]. In this study, the mechanisms of mild head injury and moderate risk were not significantly associated with intracranial hemorrhage. Note that the mechanisms occurred in less than 10% of patients without intracranial group: speed of vehicle over 40 km/h (10.61%) and ejection from vehicles (2.99%) as shown in Table 2.

Previous studies have reported several factors to be suggestive of intracranial hemorrhage such as history of vomiting or amnesia [9–11]. However, these were not found to be independent factors in this study (Table 3). These findings may be explained by differences in study populations. In this study, we enrolled only patients aged between 15 and 59 years and who were at moderate risk for intracranial hemorrhage. This is in contrast to some previous studies, which included patients of all ages and at all levels of risk for intracranial hemorrhage [10, 11]. Elderly patients may have more symptoms of intracranial hemorrhage such as vomiting and amnesia.

Hypertension (HT) has been shown to double the risk for intracerebral and subarachnoid hemorrhage [12, 13]. High blood pressure directly causes narrowing and occlusion of the small blood vessels in the brain [14], possibly leading to a greater risk for intracranial hemorrhage in mild head injury cases. Hypertension had the highest odds ratio among independent factors at 11 times (Table 3). The hemorrhage may be enlarged in hypertensive patients, particularly in the first six hours, due to breakdown of the blood-brain barrier and dysregulation of hemostasis [15].

Another independent factor for intracranial hemorrhage is GCS. A previous study found that a reduction in GCS score from 15 to 14 in cases of mild traumatic head injury was accompanied by a fourfold greater risk for intracranial hemorrhage (95% CI of 1.72–9.80) [9]. In this study, a baseline GCS of 13 carried with it a 100% risk of intracranial hemorrhage (Figure 1). We, therefore, would like to remind the emergency physicians that in mild traumatic head injury with a GCS of 13 they should be aware of intracranial hemorrhage even without any symptoms. In other words, it would be better to categorized moderate risk of mild head injury as the GCS score of 14-15.

There were some limitations in this study. First, only those patients who underwent a CT scan of the brain were included. In addition, results of this study applied only to those who were at moderate risk for mild head injury. Note that this study was a single site study conducted in Thailand. There might be different results in other countries. Moreover, the study was based on data that were collected retrospectively. Some factors may be missing. Finally, due to the small sample size, the 95% CI may be wide such as hypertension (Table 3). We ensure the adequate power of the sample size by tracing back the study power. By the difference of proportions of loss of consciousness between the groups of with and without intracranial hemorrhage, the power of study was 99.6%.

## 5. Conclusions

The independent factors associated with intracranial hemorrhage in patients with mild head injury who were determined to be at moderate risk for the condition included history of hypertension, headache, loss of consciousness, and baseline GCS score. A GCS score of 13 was associated with 100% risk for intracranial hemorrhage.

## Figures and Tables

**Table 1:** Baseline characteristics of head injury patients who were at moderate risk for intracranial hemorrhage categorized by evidence of intracranial hemorrhage from computed tomography of the brain.

Factors	No intracranial hemorrhage *n* = 135	Intracranial hemorrhage *n* = 18	*p* value
Mean (SD) age, years	37.59 (1.16)	41.44 (2.82)	0.247
Male gender	48 (35.56)	4 (22.22)	0.303
Triage level at presentation			0.116
1	3 (2.27)	0	
2	65 (49.24)	13 (72.22)	
3	54 (40.91)	4 (22.22)	
4	9 (6.82)	0	
5	1 (0.76)	1 (5.56)	
Mean (SD) Glasgow Coma Scale score	14.85 (0.47)	14.39 (0.85)	<0.001
Comorbidities			
Diabetes mellitus	10 (7.41)	3 (16.67)	0.183
Hypertension	9 (6.67)	4 (22.22)	0.049
Stroke	4 (2.96)	0	0.999
Coronary artery disease	4 (2.96)	0	0.999
Medications			
NSAIDs	4 (2.96)	0	0.999
Aspirin	4 (2.96)	2 (11.11)	0.148
Clopidogrel	3 (2.22)	2 (11.11)	0.106
Warfarin	1 (0.74)	0	0.999

*Note*. Data presented as number (percentage) unless indicated otherwise; NSAIDs: nonsteroidal anti-inflammatory drugs.

**Table 2:** Symptoms/signs and characteristics of head injury patients who were at moderate risk for intracranial hemorrhage categorized by evidence of intracranial hemorrhage from computed tomography of the brain.

Factors	No intracranial hemorrhage *n* = 135	Intracranial hemorrhage *n* = 18	*p* value
Causes of head injury			
Motorcycle	44 (32.59)	3 (16.67)	0.276
Car	10 (7.41)	0	0.608
Falling	52 (38.81)	8 (44.44)	0.798
Assault	8 (5.93)	1 (5.56)	0.999
Symptoms and signs			
Syncope	12 (8.89)	5 (27.78)	0.032
Headache	33 (24.63)	9 (50.00)	0.045
Amnesia	47 (34.81)	10 (55.56)	0.119
Loss of consciousness	36 (26.67)	14 (77.78)	<0.001
Large facial wound	11 (8.15)	0	0.363
Other injuries	17 (12.59)	0	0.224
Characteristics of head injuries			
Speed > 40 km/h	14 (10.61)	0	0.220
Ejection from vehicles	4 (2.99)	0	0.999
Alcohol intoxication	19 (14.07)	0	0.130

*Note*. Data presented as number (percentage); VAS: visual analogue scale out of 10.

**Table 3:** Significant factors associated with intracerebral hemorrhage as diagnosed using computed tomography of the brain in head injury patients who were moderate risk for intracranial hemorrhage.

Factors	Unadjusted odds ratio (95% confidence interval)	Adjusted odds ratio (95% confidence interval)
Diabetes mellitus	2.500 (0.618, 10.107)	0.617 (0.084, 4.621)
**Hypertension**	**4.000 (1.089, 14.689)**	**11.376 (1.317, 98.262)**
Motorcycle	0.414 (0.114, 1.504)	0.522 (0.116, 2.354)
Syncope	3.942 (1.200, 12.953)	1.043 (0.235, 4.630)
**Headache**	**3.051 (1.121, 8.353)**	**4.011 (1.097, 14.661)**
Amnesia	2.340 (0.865, 6.329)	1.908 (0.500, 7.282)
**Loss of consciousness**	**9.625 (2.973, 31.162)**	**10.282 (2.436, 43.395)**
**Glasgow Coma Scale score**	**0.350 (0.179, 0.685)**	**0.164 (0.057, 0.474)**

*Note*. Bold indicated significant factors.

## References

[1] Faul M. X. L., Wal M. M., Coronado V. G. (2010). *Traumatic Brain Injury in the United States: Emergency Department Visits, Hospitalizations, and Deaths 2002–2006*.

[2] Levin H. S., Narayan R. K., Wilberger J. E., Povlishock J. T. Outcome from mild head injury. *Neurotrauma*.

[3] Haydel M. J., Preston C. A., Mills T. J., Luber S., Blaudeau E., DeBlieux P. M. C. (2000). Indications for computed tomography in patients with minor head injury. *The New England Journal of Medicine*.

[4] Stiell I. G., Clement C. M., Rowe B. H. (2005). Comparison of the Canadian CT head rule and the New Orleans criteria in patients with minor head injury. *Journal of the American Medical Association*.

[5] (2007). Summaries for patients. Predicting intracranial traumatic findings on computed tomography in patients with minor head injury: the CHIP prediction rule. *Annals of Internal Medicine*.

[6] Kuppermann N., Holmes J. F., Dayan P. S. (2009). Identification of children at very low risk of clinically –important brain injury after head trauma: a prospective cohort study. *The Lancet*.

[7] Brenner D. J. (2002). Estimating cancer risks from pediatric CT: going from the qualitative to the quantitative. *Pediatric Radiology*.

[8] Yuksen C., Sawatmongkornkul S., Suttabuth S., Sawanyawisuth K., Sittichanbuncha Y. (2016). Emergency severity index compared with 4-level triage at the emergency department of Ramathibodi University Hospital. *Asian Biomedicine*.

[9] Ibanez L., Chan S., Silva J. (2004). Reliability of clinical guidelines in the detection of patients at risk following mild head injury: Results of a prospective study. *Journal of Neurosurgery*.

[10] Smits M., Diederik W., Dippel W. (2007). Predicting intracranial traumatic findings on computed tomography in patients with minor head injury: the CHIP prediction rule. *Annals of Internal Medicine*.

[11] Fabbri A., Servadei F., Marchesini G. (2005). Clinical performance of NICE recommendations versus NCWFNS proposal in patients with mild head injury. *Journal of Neurotrauma*.

[12] Chobanian A. V., Bakris G. L., Black H. R. (2003). The seventh report of the joint national committee on prevention, detection, evaluation, and treatment of high blood pressure: the JNC 7 report. *The Journal of the American Medical Association*.

[13] Thrift A. G., McNeil J. J., Forbes A., Donnan G. A. (1996). Risk factors for cerebral hemorrhage in the ear of well-controlled hypertension. *Stroke*.

[14] Garcia J. H., Ho K. L. (1992). Pathology of hypertensive arteriopathy. *Neurosurgery Clinics of North America*.

[15] Balami J. S., Buchan A. M. (2012). Complications of intracerebral haemorrhage. *The Lancet Neurology*.

